# Transcriptome analysis reveals the regulatory mechanism of myofiber development in male and female black Muscovy duck at different ages

**DOI:** 10.3389/fvets.2024.1484102

**Published:** 2024-11-20

**Authors:** Weihong Zhang, Mengyun Zou, Xiaolan Xiong, Yue Wei, Changling Ke, Haiqin Li, Jinfang Xie, Qipeng Wei, Jiangnan Huang

**Affiliations:** ^1^Institute of Animal Husbandry and Veterinary Medicine, Jiangxi Academy of Agricultural Sciences, Jiangxi Poultry Engineering Technology Research Center, Jiangxi Poultry Breeding Engineering Laboratory, Nanchang, Jiangxi, China; ^2^Jiujiang Academy of Agricultural Sciences, Jiujiang, Jiangxi, China

**Keywords:** black Muscovy duck, myofiber, RNA-seq, meat quality, muscle development

## Abstract

**Introduction:**

Sexual dimorphism in Muscovy ducks results in substantial differences in muscle development potential between males and females, leading to significant variations in growth rates and body weights throughout their development.

**Methods:**

This study aimed to investigate the regulatory mechanisms underlying the differences in muscle development between genders in black Muscovy ducks, we analyzed the phenotypic characteristics and transcriptome profiles of breast muscles in male and female black Muscovy ducks at different developmental stages (postnatal days 28, 42, and 70).

**Results:**

In the analysis of tissue physical morphology, the results showed that females exhibit larger myofiber diameters and lower myofiber densities compared to males at postnatal day 42 (*p* < 0.05). The difference becomes more pronounced by day 70, however, no significant difference was observed at postnatal day 28. Transcriptome analysis identified a total of 1,118 unique differentially expressed genes (DEGs) across the various comparison groups. In different growth and development stages of black Muscovy ducks, the DEGs like *MYLK4*, *KIT*, *CD36*, *ATP2A1* were significantly associated with myofiber hypertrophy, and key pathways such as AMPK signaling pathway, focal adhesion, and ECM-receptor interactions have been found to be closely associated with muscle size and hypertrophy. In the breast muscles of different sexes black Muscovy ducks, the DEGs such as *TPM2*, *HNRNPK*, *VCP*, *ATP2A2*, and *ANKRD1* may be the reason for the difference in breast muscle size between male and female ducks. Furthermore, key pathways, including the cGMP-PKG signaling pathway, calcium signaling pathway, and hypertrophic cardiomyopathy are also involved in regulating the developmental potential differences in muscle between male and female ducks.

**Discussion:**

This study reveals the molecular mechanism regulating the muscle development in male and female black Muscovy ducks at different growth stages, and provides valuable insights into the specific genes responsible for muscle development, laying a theoretical foundation for enhancing the genetic quality of duck meat.

## 1 Introduction

Muscovy ducks is recognized worldwide as an excellent lean meat duck breed, characterized by its large body weight, high lean meat yield, and rapid growth, and has a valuable and uniquely positioned within the modern poultry industry (1). Muscovy ducks showed significant sexual dimorphism in terms of their body weight, and males grow much faster than female (2). Notably, previous study found that there was no significant difference in body weight between the male and female Muscovy ducks before 28 days of age, with growth rates for male and female ducks being relatively similar during this period. Interestingly, after 28 days of age, the growth rate accelerate significantly, with male ducks growing faster than females, and the growth peaks at 42 days of age and stabilization at 70 days of age (3). Therefore, exploring muscle development differences among ducks of various sexes and ages, enhances understanding of developmental mechanisms and improves livestock production efficiency.

Myofibers, also known as myoblasts, originate from embryonic mesoderm and are the basic units of muscle, and their number, diameter, and density are closely related to meat yield and meat quality (4). Postnatal myofiber growth occurs through muscle fiber enlargement by the process of hypertrophy, which results from the recruitment of satellite cell nuclei (5). Nevertheless, there is limited research on the relationship between myofiber development and muscle growth in poultry. Huo et al. evaluated the myofiber characteristics of fast-growing and slow-growing ducks, and found that there were significant differences in fiber diameter, fiber density, and fiber cross-sectional area (CSA) between duck breeds with different growth rates, and that meat quality traits, such as intramuscular fat content, were also significantly affected by breeds (6). It is well known that the growth and development of muscle are influenced by a complex regulatory network. Xu et al. successfully analyzed the differential expression of pectoral muscle and subcutaneous adipose tissue in Peking ducks, identifying genes associated with muscle development and fat deposition (7). Additionally, potential candidate genes and signaling pathways related to body size and carcass traits in Peking ducks were identified (8).

Although functional genes associated with muscle development in ducks have been discovered, more in-depth and systematic studies are needed to comprehensively reveal the molecular mechanisms underlying the muscle differential development of ducks across different sexes and ages. In recent years, RNA-seq has been widely used in study on poultry transcriptomes. Which is helpful to reveal new genes, as well as the pathways. Although the detection accuracy of traditional Illumina RNA-seq is high, its reads are limited in length and the spliced full-length transcripts are incomplete. In contrast, Iso-Seq can directly obtain full-length transcript sequences and detect multiple variable splicing forms, which can make up for the shortcomings of Illumina RNA-seq, thereby maximizing the reliability and comprehensiveness of transcriptome information (9, 10). In this study, we performed full-length transcriptome sequencing (PacBio Iso-Seq) and Illumina RNA-seq of male and female Muscovy duck muscle samples at three developmental stages (D28, D42, and D70) to identify the DEGs and signaling pathways involved in myofiber development in Muscovy duck, and to analyze the correlation of these DEGs with the myofiber phenotype. This study makes a valuable contribution to the existing knowledge related to black Muscovy duck species and lays a theoretical foundation for the development and utilization of their genetic resources. This study makes a valuable contribution to the existing knowledge related to black Muscovy duck species and lays a theoretical foundation for the development and utilization of their genetic resources.

## 2 Materials and methods

### 2.1 Ethics statement

The Ministry of Agriculture of China’s Guidelines for the Care and Use of Experimental Animals were strictly adhered to during all animal procedures. The protocols conducted in this study were approved by the Animal Ethics Committee of the Institute of Animal Husbandry and Veterinary, Jiangxi Academy of Agricultural Science (JXAAS 2020-0025).

### 2.2 Animals and sample collection

The experimental subjects consisted of 600 black Muscovy ducks that hatched simultaneously, and the hatched eggs were produced by a 300-day-old black Muscovy duck population. All the ducks were divided into male and female groups for feeding (female: 320; male: 280). Tissue samples were obtained from black Muscovy ducks at three different ages: 28, 42, and 70 days. Black Muscovy ducks in each age were divided into male and female groups, and each group consisted of three replicates, with two breast muscle samples collected in each replicate. Each sample was extracted from the same region and portion of the right breast major muscle using forceps and scissors, with a moderate size. Among them, the sample size used for tissue morphology detection is 1 cm * 1 cm * 1 cm (length * width * height) muscle blocks. Thus, a total of 18 breast muscle samples were collected for RNA sequencing, 18 breast muscle samples were collected for muscle histomorphometry, and all breast muscle samples were carefully sealed and appropriately labeled.

### 2.3 Total RNA extraction and quality testing

To extract total RNA from the breast tissue, TRIzol reagent (Invitrogen, Waltham, MS, USA) was used according to the manufacturer’s instructions. The integrity and quality of the RNA were assessed by running electrophoresis on 1% agarose gels, while the RNA concentration was further determined utilizing an RNA Nanop2000 (Thermo Scientific, CA, USA) to acquire RNA samples that fulfilled the requirements of both qPCR and RNA-seq analyses. Subsequently, cDNA was synthesized using a RevertAid First Strand cDNA Synthesis Kit (#K1622; Thermo Scientific, Wilmington, DE, USA) following the protocol outlined by the manufacturer. To maintain their stability, all remaining tissue samples, RNA, and cDNA were stored in an ultralow temperature refrigerator at −80°C.

### 2.4 Library preparation and sequencing

To prepare the Iso-Seq library, we followed the protocol provided by Pacific Biosciences (PN 100-092-800-03) and used a Clontech SMARTer PCR cDNA Synthesis Kit to perform isoform sequencing (Iso-Seq). We then analyzed the sequencing results using bioinformatic tools and processed the sequence data using SMRT Link 6.0 software. Iso-Seq can directly obtain full-length transcript sequences and detect multiple variable splicing forms. To correct any additional nucleotide errors in the consensus reads, we utilized LoRDEC software along with the Illumina RNA-seq data (11). Illumina RNA-seq improves the sensitivity of sequence detection, thereby maximizing the reliability and comprehensiveness of transcriptome information. Moreover, we employed CD-HIT to remove any redundancy in the corrected consensus reads, resulting in the generation of final transcripts for subsequent analysis (12).

### 2.5 Screening of DEGs and enrichment analysis

We employed the DESeq R package (version 1.10.1) to conduct differential expression analyses between two conditions or groups. DESeq employs statistical methods based on the negative binomial distribution to identify differential expression in digital gene expression data. To control for false discoveries, we adjusted the resulting *p* values using the Benjamini–Hochberg method. Genes with an adjusted *p* value <0.05 and an absolute log2-fold change (|log2FC|) > 1, as determined by DESeq, were identified as differentially expressed genes (13). We used “BM28M,” “BM28F,” “BM42M,” “BM42M,” “BM70F,” and “BM70F” to represent “28-day-old male black Muscovy ducks,” “28-day-old female black Muscovy ducks,” “42-day-old male black Muscovy ducks,” “42-day-old female black Muscovy ducks,” “70-day-old male black Muscovy ducks,” and “70-day-old female black Muscovy ducks,” respectively.

To perform a Gene Ontology (GO) enrichment analysis of the DEGs, we utilized the GOseq R package, which corrects for gene length bias and ensures fair gene reads to accurately measure gene expression. GO terms with corrected *p* values less than 0.05 were considered to be significantly enriched among the DEGs. To gain insights into the high-level functions and biological utilities of the system at the molecular level, including the cell, organism, and ecosystem, we leveraged molecular-level information, particularly large-scale molecular datasets generated via genome sequencing and other high-throughput experimental technologies. For this purpose, we utilized the Kyoto Encyclopedia of Genes and Genomes (KEGG) database resource to detect the statistical enrichment of DEGs in KEGG pathways via KOBAS 3.0 software.^1^

### 2.6 Validation of sequencing results by real-time PCR

qPCR was used to detect the relative expression levels of eleven DEGs. qPCR was conducted on an Archimed Quantitative PCR Detection System using SYBR® Select Master Mix (2X) (4,472,908; ABI, Waltham, MA, USA). The reference gene used was HPRT1, and the primer sequences can be found in Supplementary Table A1. Each sample was repeated three times. The 2^−∆∆CT^ method was applied to normalize the qPCR results, after which the normalized data were subjected to statistical analysis. *p* < 0.05 was considered to indicate a significant difference. The results of the data analysis were plotted using GraphPad Prism 9.0 (GraphPad Software, USA).

### 2.7 Sections production

When the breast muscle tissue was fixed by tissue fixative (formalin solution), we sequentially dehydrated, transparent and paraffin embedded the breast muscle tissue, and then trimmed and sectioned the embedded wax block, and finally stained it by hematoxylin–eosin, as for the observation and photographing under the microscope, all the actual operations were done by Wuhan Saiweier Biotechnology Co., Ltd.

## 3 Results

### 3.1 Physical morphology of duck breast muscle

**Figure 1:**
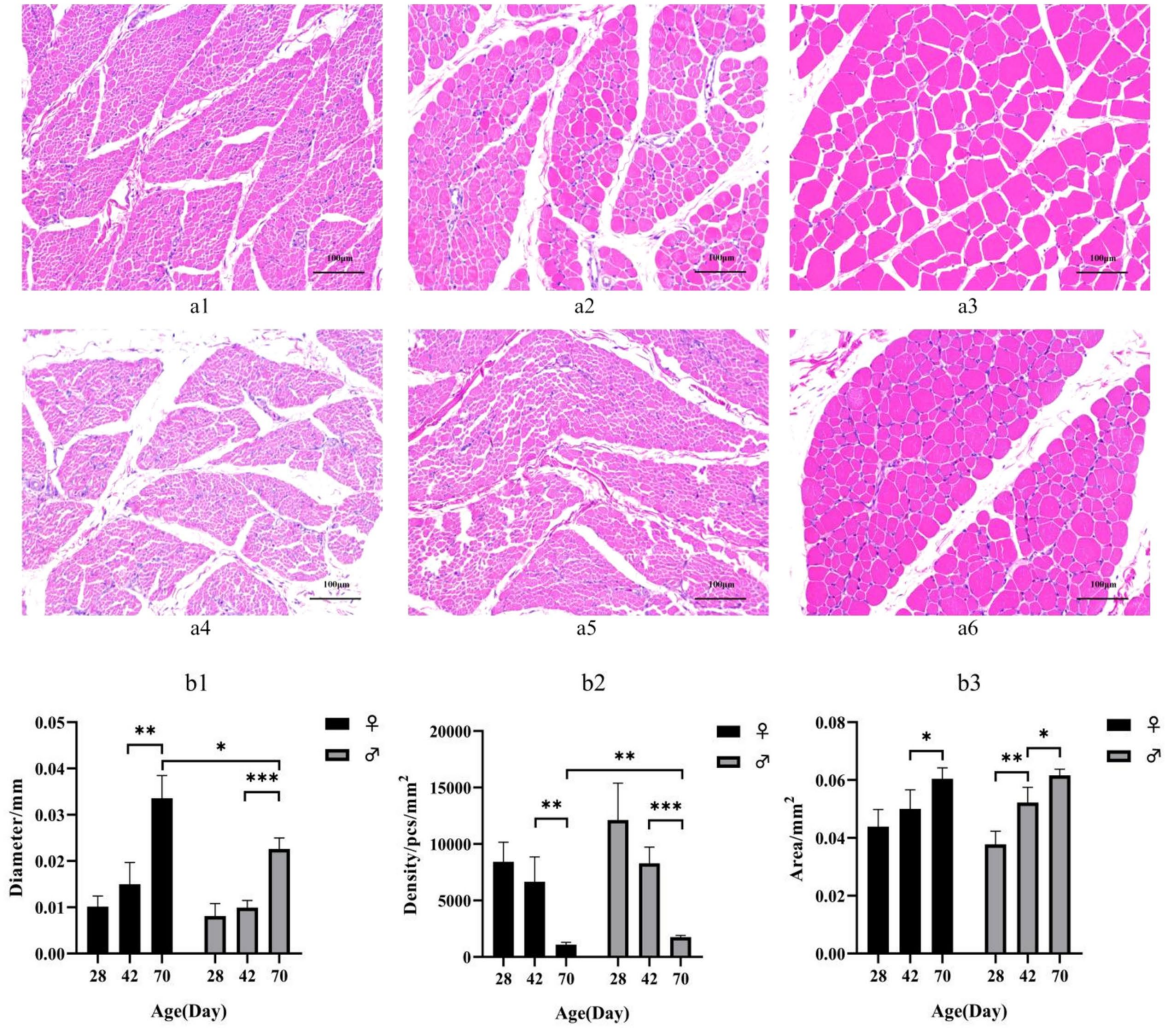
**(a)** HE-stained sections of breast muscle of different sexes and different day-age segments. **(a1)** represents 28-day-old female individuals, **(a1)** represents 42-day-old female individuals, **(a2)** represents 70-day-old female individuals, **(a3)** represents 28-day-old male individuals, **(a4)** represents 42-day-old male individuals and (a5) represents 70-day-old male individuals. **(b)** Characteristics of myofiber in Muscovy ducks of different sexes and different ages. Mean + SD, *n* = 4; **(b1)** represents Myofiber diameter, **(b2)** represents myofiber density, and **(b3)** represents myofiber cross-sectional area. * indicates *p* < 0.05, ** indicates *p* < 0.01, *** indicates *p* < 0.001.

HE staining showed that myofibril diameter increased and density decreased with increasing age (Figure 1a). Females exhibit larger myofiber diameters and lower myofiber densities compared to males at postnatal day 42. The difference becomes more pronounced by day 70, the myofiber diameters in female ducks was larger than that in males (*p* < 0.05), while the myofiber densities was highly significant smaller than that in males (*p* < 0.01). However, no significant difference was observed at postnatal day 28 in the analysis of tissue physical morphology (Figure 1b).

### 3.2 Transcriptome overview

In this study, the comparison rates of reads for all samples ranged from 75 to 82% (Table 1), with Q20 above 97%, Q30 above 95%, and GC content between 49 and 52%. A total of 171,439 transcripts were detected through transcriptome sequencing. After correction and redundancy removal, 109,718 clean data points were obtained. Among these, 88,204 transcripts (over 80% of the total) were unique (Table 2). Functional gene annotation was performed on all transcripts using five databases (Nt, Nr, KOG/COG, GO, and KEGG), resulting in 30,762 common transcript annotations (Supplementary Figure A1).

**Table 1:** List of raw data quality control analysis.

Sample name	Raw reads	Clean reads	Mapped reads	Q20 (%)	Q30 (%)	GC content (%)
BM28_F_1	52,575,786	51,524,270	41,174,804 (79.91%)	98.54	96.54	49.75
BM28_F_2	69,229,953	67,153,054	52,841,878 (78.69%)	98.63	96.21	50.35
BM28_F_3	64,980,945	63,681,326	51,416,066 (80.74%)	98.76	96.79	49.54
BM28_M_1	55,469,058	53,804,986	42,226,982 (78.48%)	98.23	96.33	49.36
BM28_M_2	56,199,169	54,400,796	42,420,686 (77.98%)	97.22	95.21	50.95
BM28_M_3	69,814,363	68,418,076	55,336,712 (80.88%)	98.43	96.89	49.72
BM42_F_1	55,729,287	54,893,348	43,948,352 (80.06%)	98.55	95.55	49.74
BM42_F_2	46,634,510	45,701,820	36,647,056 (80.19%)	97.99	96.09	49.99
BM42_F_3	47,904,362	46,802,562	35,330,508 (75.49%)	98.14	95.74	50.32
BM42_M_1	55,815,412	54,475,842	43,628,098 (80.09%)	97.98	95.01	49.89
BM42_M_2	50,793,163	49,523,334	39,020,052 (78.79%)	99.01	96.78	51.15
BM42_M_3	55,034,343	53,603,450	42,333,876 (78.98%)	98.39	95.49	51.43
BM70_F_1	56,305,858	55,348,658	45,488,528 (82.19%)	98.11	95.21	52.18
BM70_F_2	63,018,673	61,758,300	49,703,168 (80.48%)	98.22	95.72	49.79
BM70_F_3	67,899,629	66,609,536	54,500,252 (81.82%)	98.18	95.87	49.98
BM70_M_1	56,529,785	55,851,428	44,345,652 (79.40%)	98.29	95.13	50.12
BM70_M_2	52,317,772	50,382,014	40,597,186 (80.58%)	98.31	95.21	51.34
BM70_M_3	55,849,623	54,285,834	43,707,108 (80.51%)	97.72	95.31	51.44

**Table 2:** List of transcript numbers.

Sample	Total_number of transcripts	Number of clean transcripts	Unique number
Breast muscle	171,439	109,718	88,204

### 3.3 Screening and analysis of differential genes

**Figure 2:**
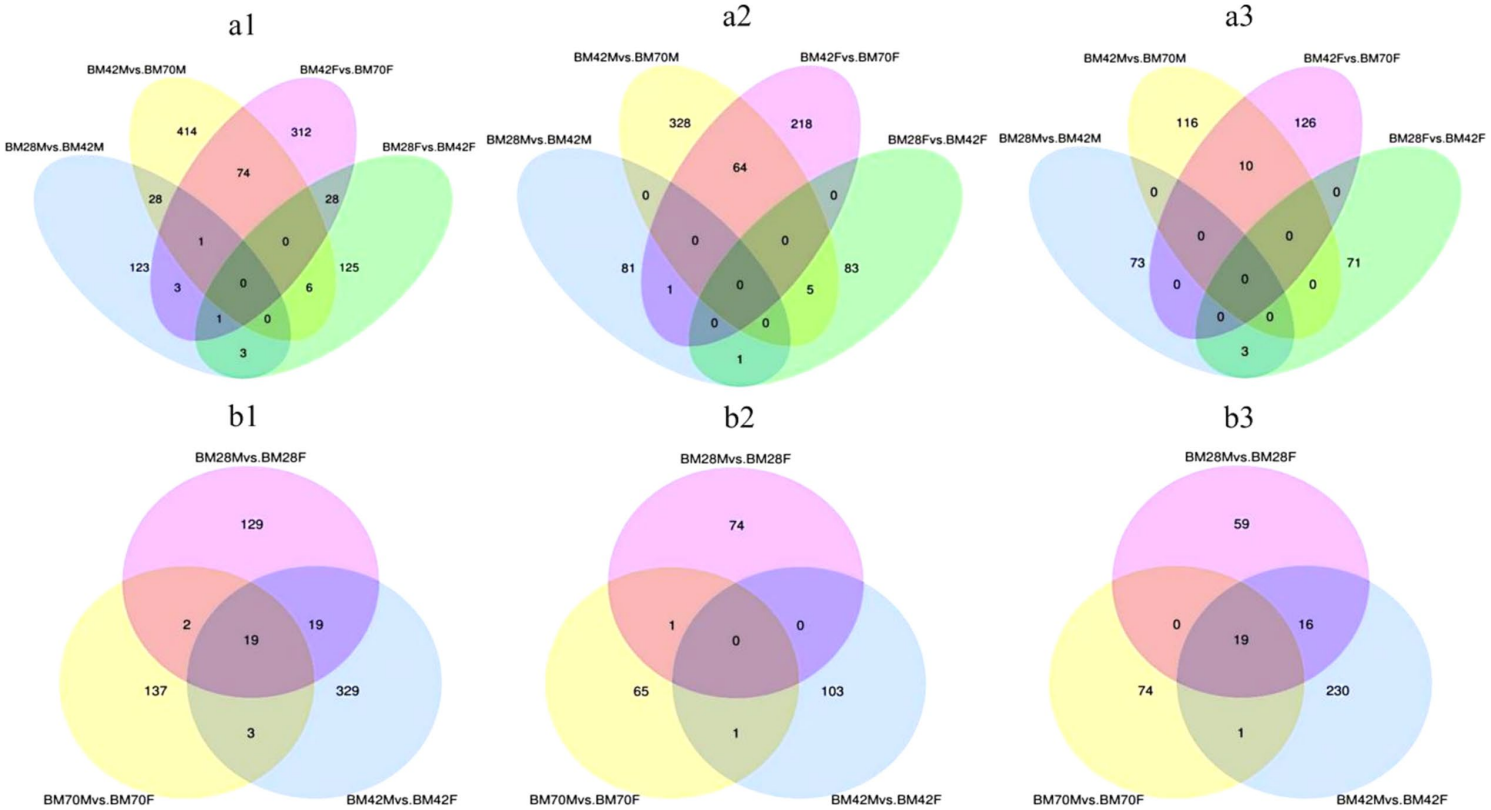
**(a)** Venn diagrams of DEGs for the same sex and different age groups. **(a1)** represents the Venn diagram of all differential genes, **(a2)** represents the Venn diagram of up-regulated differential genes, and **(a3)** represents the Venn diagram of down-regulated genes. **(b)** Venn diagrams of DEGs among different sex groups at the same age. **(b1)** represents the Venn diagram of all DEGs, **(b2)** represents the Venn diagram of up-regulated DEGs, and **(b3)** represents the Venn diagram of down-regulated genes. The sum of the numbers in each large circle represents the total number of genes expressed in the sample/group, and the overlapping parts of the circles represent the expressed genes shared between samples/groups.

Within the same sex and between the different age groups (Figure 2a and Table 3), we screened a total of 1,118 unique DEGs, including 653 DEGs in the male duck group and 553 DEGs in the female duck group. In the BM28M vs. BM42M comparison, a total of 159 DEGs were detected, consisting of 83 upregulated genes and 76 downregulated genes. Similarly, the 523 DEGs were differentially expressed between the BM42M and BM70M groups, including 397 upregulated genes and 126 downregulated genes. In the BM28F vs. BM42F comparison, a total of 163 DEGs were detected, with 89 upregulated genes and 74 downregulated genes. In the BM42F vs. BM70F comparison, a total of 419 DEGs were identified, 283 of which were upregulated, while 136 were downregulated.

**Table 3:** The number of DEGs in different groups.

Comparison group	Total DEGs	Up	Down
BM28F vs. BM42F	163	89	74
BM42F vs. BM70F	419	283	136
BM28M vs. BM42M	159	83	76
BM42M vs. BM70M	523	397	126
BM28F vs. BM28M	169	75	94
BM42F vs. BM42M	370	104	266
BM70F vs. BM70M	161	67	94

The vertical coordinates represent comparison groups. BM-Black Muscovy; 28,42,70-day-old; F/M- female/male, as described below.

The Venn diagram (Figure 2a) illustrates that only four candidate genes exhibited co-differential expression in male and female black Muscovy ducks from 28 to 42 days of age. These genes included *COL20A1*, *HNRNPA2B1*, and *PALD1*, which were up-regulated, and *WSB1*, which was down-regulated (Supplementary Table A7). Furthermore, 75 DEGs were identified as co-differently expressed in male and female black Muscovy ducks from 42 days to 70 days of age. This group included down-regulated genes (e.g., *ABLIM3*, *MAT1A*, *COL1A1*, *COL1A2*), alongside up-regulated genes (e.g., *RNF168*, *SLC38A4*) (Supplementary Table A8). In female ducks, 29 DEGs exhibited sustained significant differences across three developmental stages: D28, D42 and D70. Among these, 20 DEGs were down-regulated from D28 to D42 and then up-regulated (e.g., *MYBPC1*, *RBM5*, *UACA*), while 9 DEGs were up-regulated D28 to D42 and then down-regulated (e.g., *PLXND1*, *PAICS*, *MAF1*, *TMOD1*) (Supplementary Table A4). Similarly, in male ducks, 29 DEGs demonstrated significant differences across the same three stages, with 21 DEGs initially down-regulated and subsequently up-regulated (e.g., *FSTL1*, *PGM5*, *MSRB1*), and 8 DEGs that were first up-regulated and then down-regulated (e.g., *TGFB3*, *LANCL1*, *WTAP*) (Supplementary Table A5). However, no common genes were identified that consistently and significantly differed across the three ages in both males and females.

In both the male and female comparison groups at the same age (Figure 2b and Table 3), we identified a total of 638 DEGs. At 28 days of age (BM28F vs. BM28M), 169 differential candidate genes were detected, 75 of which were upregulated and 94 of which were downregulated. Similarly, at 42 days of age (BM42F vs. BM42M), 370 differential candidate genes were detected, with 104 genes upregulated and 266 genes downregulated. Furthermore, at 70 days of age (BM70F vs. BM70M), 161 differential candidate genes were identified, with 67 genes exhibiting upregulation and 94 genes showing downregulation. Across all the comparison groups, 19 common DEGs were identified, all of which are downregulated genes, including *HNRNPK*, *TPM2*, *ATP5A1*, *VCP*, etc. (Supplementary Table A6).

### 3.4 Differential gene enrichment

**Figure 3:**
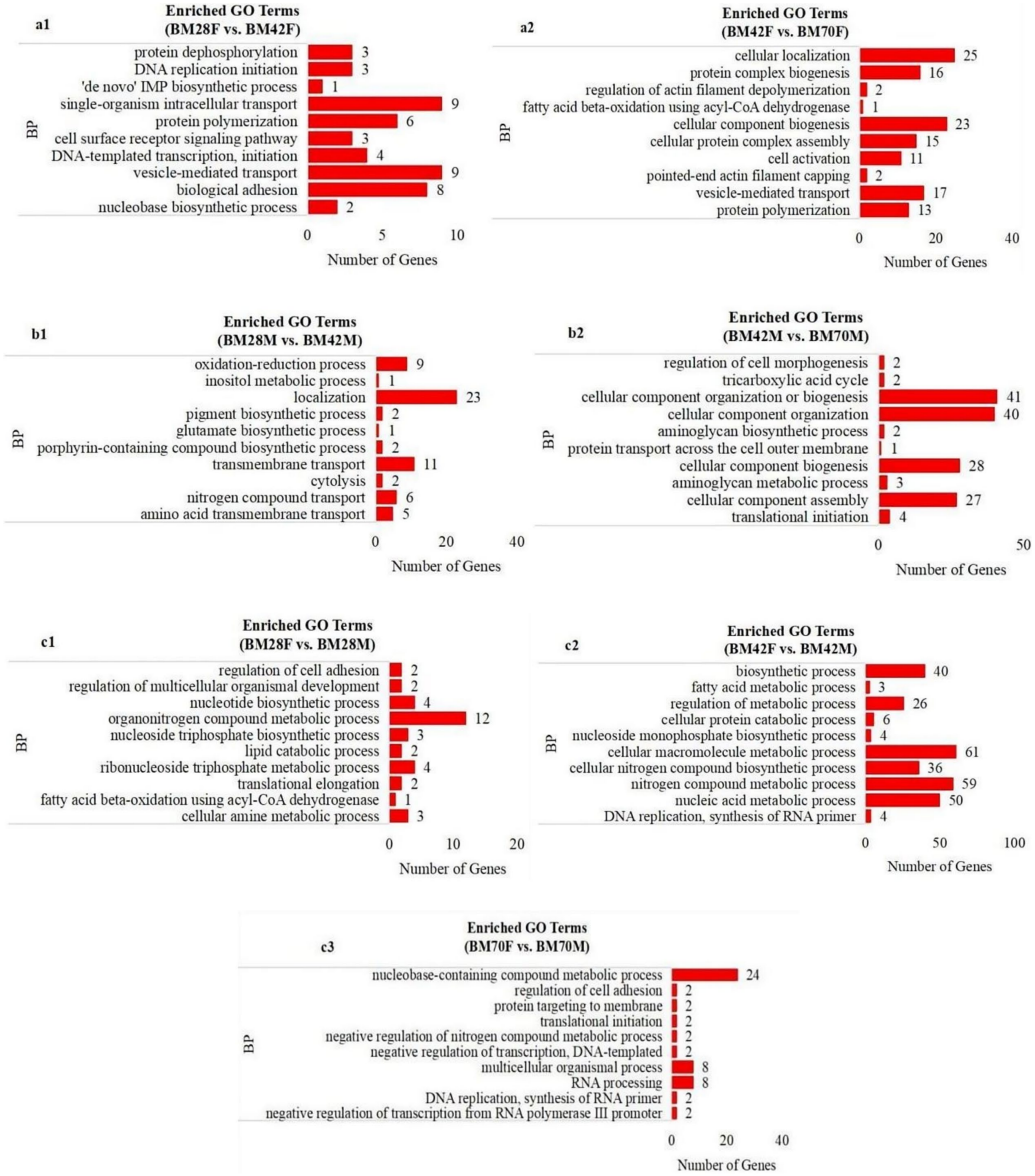
The enrichment analysis of GO functional significance. **(a1)**: BM28F vs. BM42F, **(a2)**: BM42F vs. BM70F; **(b1)**: BM28M vs. BM42M, **(b2)**: BM42M vs. BM70M; **(c1)**: BM28M vs. BM28F, **(c2)**: BM42M vs. BM42F, **(c3)**: BM70M vs. BM70F. BP-biosynthetic processes, BM-black Muscovy duck, F-female, M-male.

GO enrichment analysis revealed that the biological process terms enriched in the same sex and in different age groups were involved mainly in biosynthetic processes, such as transmembrane transport (*CACNA1S*, *TNIP1*, *NFRKB*, *SLC39A14*, *ITSN2*), protein polymerization (*TPM2*, *TMOD1*, *MyHC*), and cell component synthesis (*SLMAP*, *UNK*, *DCTN1*, *MYH1F*, *COL1A2*, *TMOD1*, *DES*) (Figures 3a,b). Conversely, metabolic processes such as fatty acid metabolism (*HSD17B12*, *GLYR1*) and nucleic acid metabolism (*SEC31A*, *SLMAP*, *PRPF3*) were predominantly enriched at the same age in different sex groups (Figure 3c).

**Figure 4:**
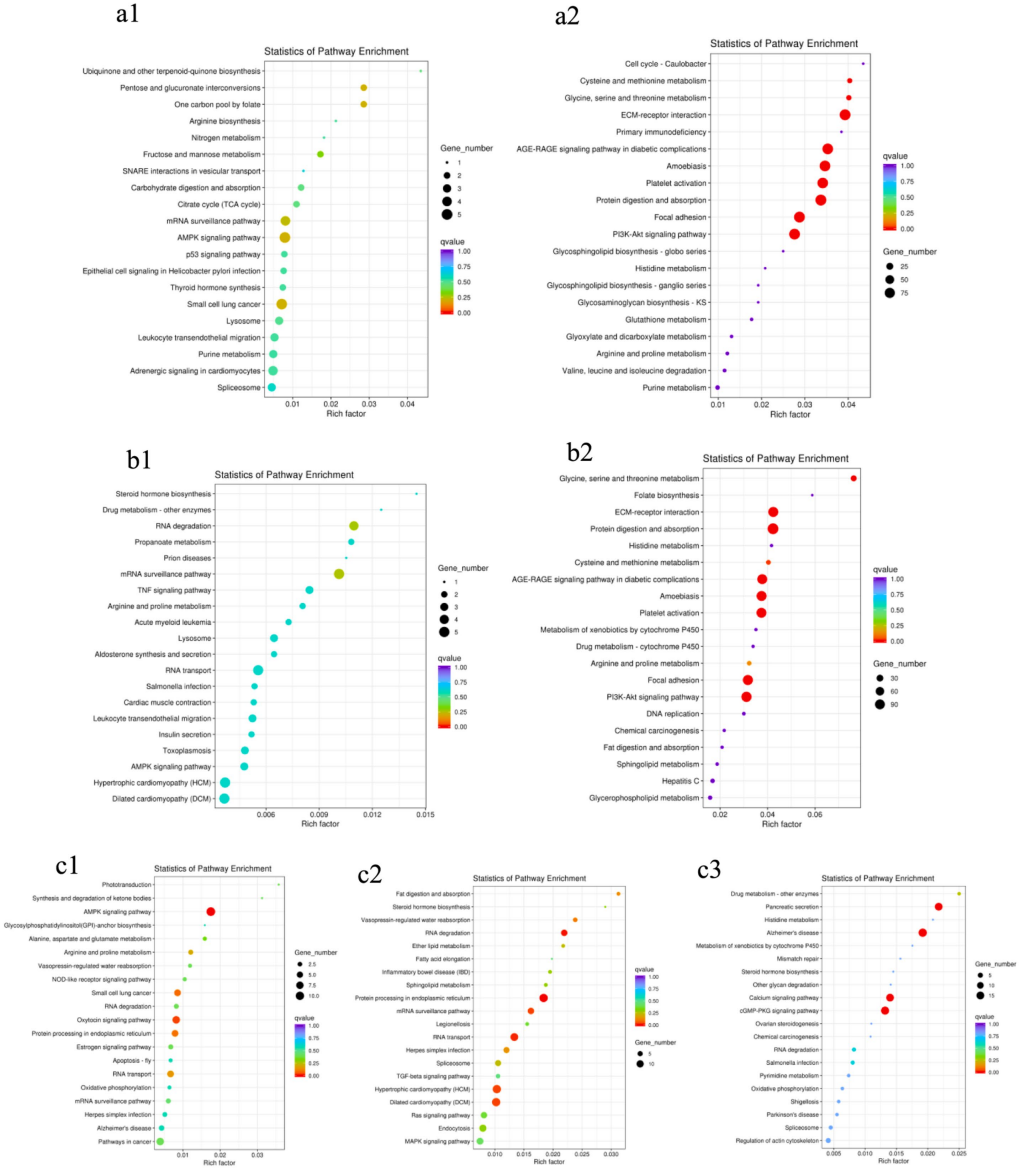
KEGG pathway enrichment scatter plots. **(a1)**: BM28F vs. BM42F, **(a2)**: BM42F vs. BM70F; **(b1)**: BM28M vs. BM42M, **(b2)**: BM42M vs. BM70M; **(c1)**: BM28M vs. BM28F, **(c2)**: BM42M vs. BM42F, **(c3)**: BM70M vs. BM70F. The vertical axis indicates the pathway name, the horizontal axis indicates the Rich factor corresponding to the pathway, the size of the *p* value is indicated by the color of the dot (the smaller the *p* value is, the closer the color is to red), and the number of DEGs in each pathway is expressed in terms of point size.

As shown in Figure 4, no significantly enriched pathways were found in the two groups of male or female ducks of different ages: BM28F vs. BM42F and BM28M vs. BM42M (Figures 4a1,b1). However, in the BM42F vs. BM70F and BM42M vs. BM70M comparison groups, a considerable number of signaling pathways were enriched and fell into two categories (Figures 4a2,b2). The first category included signaling pathways associated with cell survival, proliferation, differentiation, growth, and apoptosis. The second category included pathways that affect the metabolic function of the body, such as the ECM–receptor interaction (*CD36*, *DAG1*, *COL1A1*, *COL1A2*), the AGE–RAGE signaling pathway (*TGFB3*, *COL1A2*), FAK (*MYLK4*, *ZYX*, *EGFR*, *COL4A1*), and the PI3K–Akt signaling pathway (*HSP90A*, *CREB3*, *PPP2R3*, *COL4A5*).

Compared to the different sex groups of the same age (Figure 4c), the group at 28 days of age had slightly fewer enriched pathways than the groups at 42 days of age and 70 days of age. However, all the comparison groups exhibited differential signaling pathways that affect the regulation of biological energy metabolism. The most enriched pathways were the AMPK signaling pathway (*EEF2*, *PFKM*, *RAB11B*, *TBC1D1*, *CREB3*), RNA degradation (*PFKM*, *PABPC1*, *MTR4*, *SKIV2L2*, *SKI3*, *TTC37*), the calcium signaling pathway (*ATP2A2*, *CAMK2*, *PLCD*), and the cGMP-PKG pathway (*CREB3*, *PRKG1*, *ATP2A1*, *NFATC1*), among others.

**Figure 5:**
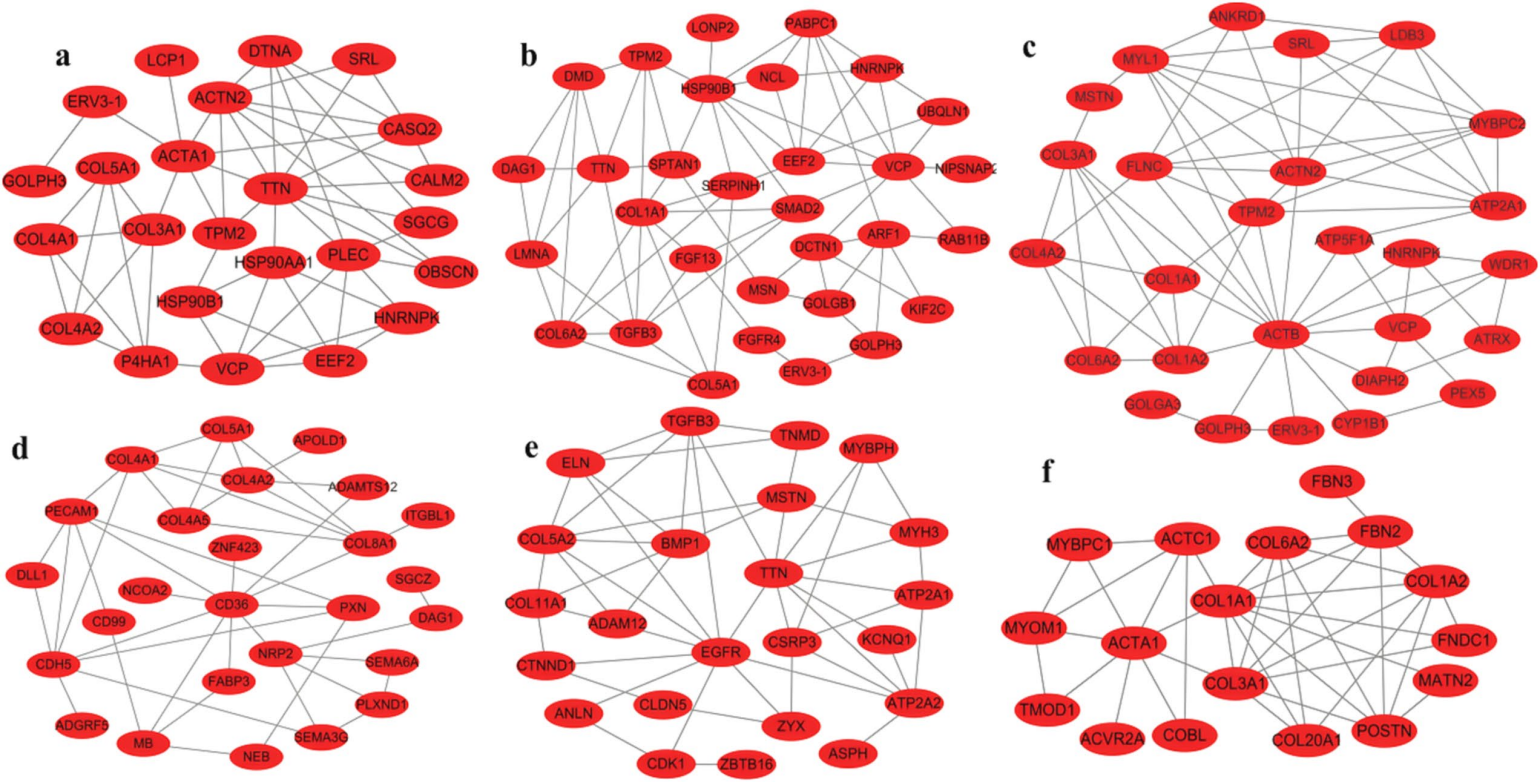
Maps of gene international network. **(a)**: Differential genes in the BM28M vs. BM28F group, **(b)**: Differential genes in the BM42M vs. BM42F group, **(c)**: Differential genes in the BM70M vs. BM70F group, **(d)**: Differential genes unique to the BM42F vs. BM70F group, compared to the BM42M vs. BM70M group, **(e)**: Differential genes unique to the BM42M vs. BM70M group, compared to the BM42F vs. BM70F group, **(f)**: Differential genes shared by BM42F vs. BM70F and BM42M vs. BM70M.

Pathway mapping revealed that DEGs such as *TPM2*, *HNRNPK*, *VCP*, *SRL*, *ERV3-1*, and collagen family genes were screened between male and female Muscovy ducks at any age group (Figures 5a–c). Since the sections showed significant differences in myofibers between 42 and 70 days of age, we specifically analyzed the DEGs in BM42F vs. BM70F and BM42M vs. BM70M groups, and found that the two groups collectively screened for genes such as *ACTA1*, *ACTC1*, *MYOM1*, and a large number of collagen family genes, which were associated with myofiber development in black Muscovy ducks (Figure 5f); DEGs such as *FABP3*, *CD36* and a large number of collagen family genes were screened in females compared to males (Figure 5d); whereas DEGs such as *ATP2A1*, *ATP2A2*, *ASPH*, *MSTN*, *TGFB3*, *ZBTB16*, *CDK1*, *COL5A2*, etc. were screened in males compared to females (Figure 5e).

### 3.5 Verification of differential expressed genes

**Figure 6:**
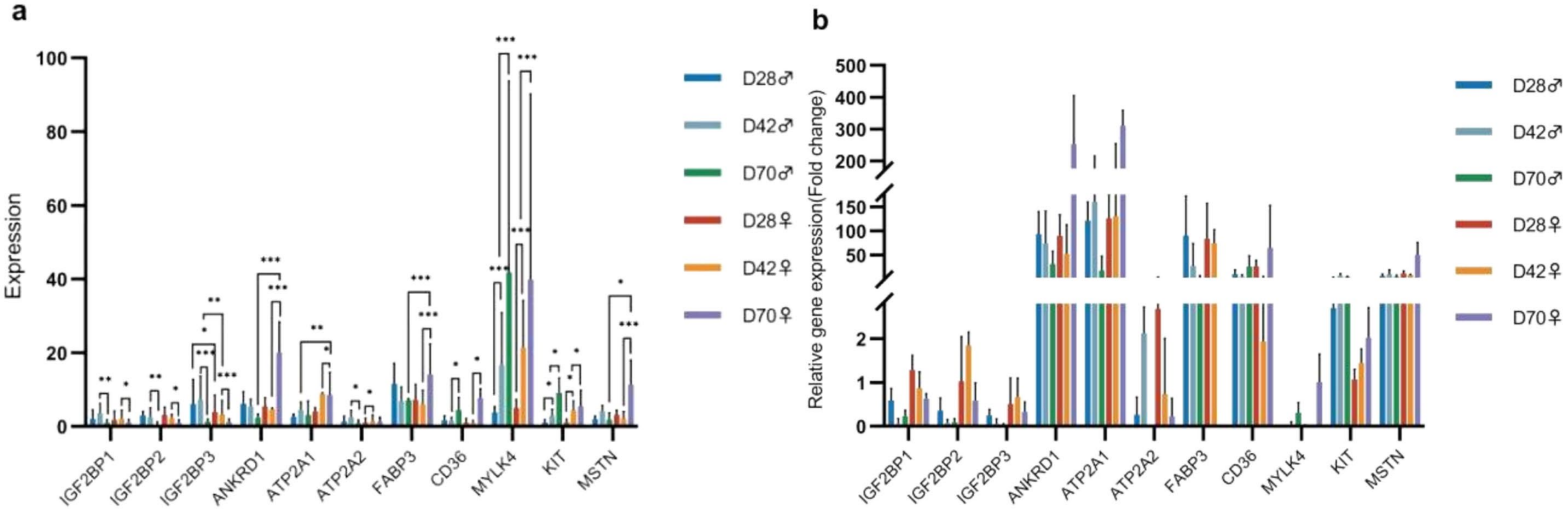
The results of differential gene expression. **(a)** Fluorescence-based quantitative PCR analysis of differentially expressed genes (Mean + SD, *n* = 3). **(b)** Verification of the sequencing data.

To validate the sequencing results, we selected 11 DEGs significant related to muscle development for qPCR (Supplementary Table A9). The results indicated that, with increasing age, the expression levels of *ATP2A2*, *MYLK4*, and *KIT* in the breast muscle of male and female black Muscovy ducks at 42 days were significantly higher than those at 28 days. In comparison to 42-days, the expression levels of *IGF2BP1*, *IGF2BP2*, and *IGF2BP3* significantly decreased (*p* < 0.05) and the expression levels of *CD36*, *MYLK4*, and *KIT* significantly increased (*p* < 0.05) in the breast muscles of both sexes at 70 days. Furthermore, at 70 days of age, the expression levels of *ANKRD1*, *FABP3*, and *MSTN* genes were significantly elevated (*p* < 0.05), whereas *ATP2A1* was significantly reduced (*p* < 0.05) in females, and *ATP2A2* expression was significantly decreased (*p* < 0.05) in males. Notably, differential gene expression between the sexes was also observed at other ages. At 28 days, the expression levels of *IGF2BP3* in females was significantly lower than that in males (*p* < 0.05). At 42 days, this trend continued, with *IGF2BP3* expression in females remaining significantly lower than in males (*p* < 0.01), while *ATP2A1* expression was significantly higher in females than in males (*p* < 0.01). The expression of these DEGs across different ages and between sexes were consistent with the trend of sequence analysis results, suggesting the reliability of the sequencing analysis (Figures 6a,b).

### 3.6 Correlation analysis

**Figure 7:**
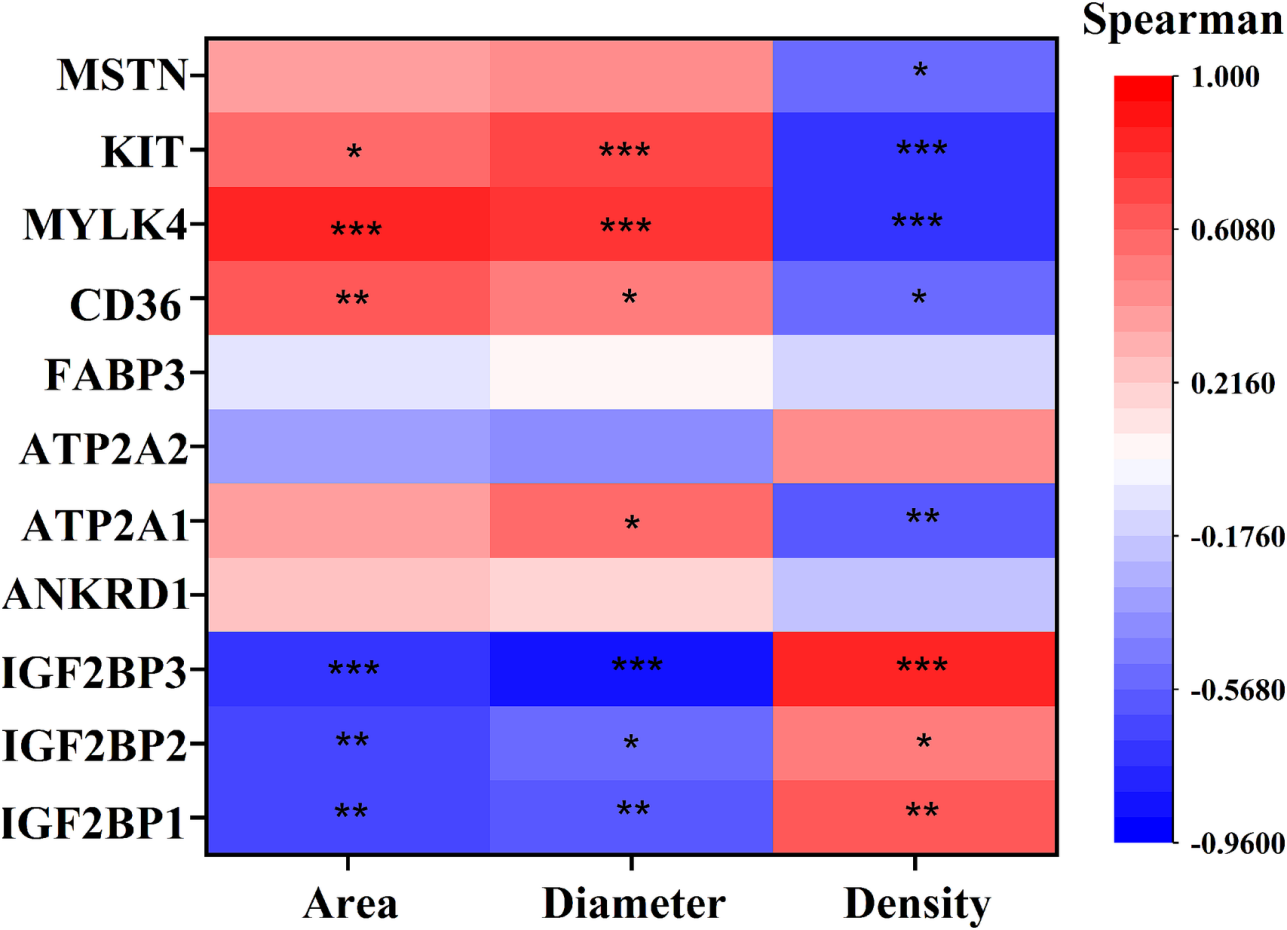
Spearman correlation analysis Graph between key DEGs and myofiber phenotypes. The vertical axis of the graph represents DEGs, and the horizontal axis is myofiber characteristics, including myofiber cross-sectional area (Area), myofiber diameter (Diameter), myofiber density (Density). The brighter the color, the stronger the correlation. * indicates *p* < 0.05, ** indicates *p* < 0.01, *** indicates *p* < 0.001.

Correlation analysis of DEGs with phenotype (Figure 7) showed that *IGF2BP1*, *IGF2BP2*, and *IGF2BP3* were significantly positively correlated with myofiber density and negatively correlated with myofiber cross-sectional area and diameter; *CD36*, *MYLK4*, and *KIT* genes were significantly negatively correlated with myofiber density and significantly positively correlated with myofiber cross-sectional area and diameter; *ATP2A1* was significantly negatively correlated with myofiber density and significantly positively correlated with myofiber diameter; and *MSTN* was significantly negatively correlated with myofiber density.

## 4 Discussion

Duck meat, as a key component of meat industry in china, occupies a pivotal position in the meat market. However, the skeletal muscle growth exhibits significant variability among different species of ducks. Muscovy ducks demonstrate superior muscle production, lower fat content, accelerated weight gain, and enhanced feed conversion efficiency compared to domestic ducks (14). Additionally, it serves as a terminal parent for producing high-quality meat ducks, specifically the Mulard duck. This characteristic renders the Muscovy duck both valuable and uniquely positioned within the contemporary poultry industry. Notably, sexual dimorphism in muscle development is evident in Muscovy ducks (2). The growth rate of male Muscovy ducks are much higher than those of female ducks after 28 days of age, and at 70 days of age, male Muscovy ducks weigh 1.5-times more than female ducks (15). In our study, the females ducks exhibit larger myofiber diameters and lower myofiber densities compared to males at 42 and 70 days of age, there was no significant difference in body weight between the male and female Muscovy ducks before 28 days of age. Numerous studies have shown that myofiber characteristics are closely related to meat tenderness, juiciness, and other quality traits, serving as important indices for measuring meat tenderness. Myofiber diameter is positively correlated with muscle shear force; larger myofiber diameters correspond to higher shear force values, resulting in decreased tenderness of duck meat (6, 16). According to previous studies, at any given age, the breast muscle of female Muscovy ducks has better flavor than that of male Muscovy ducks, but is less nutritious, tender, and juicy (17). This analysis provides us with more comprehensive information for accurately identifying differential candidate genes that contribute to the myofiber development. This is consistent with our findings that female Muscovy duck breast muscles is less tender than male Muscovy duck breast muscles.

To investigate the crucial factors necessary for the development of breast muscles in Muscovy ducks, we initially analyzed the commonly differentially expressed genes (DEGs) in both male and female black Muscovy ducks. Our investigation revealed that the expression levels of insulin-like growth factor 2 mRNA-binding proteins (*IGF2BPs*), specifically *IGF2BP1*, *IGF2BP2*, and *IGF2BP3*, remained consistently high on day 28, followed by a decline on days 42 and 70 in both male and female ducks. *IGF2BP1* has been shown to be a major gene influencing body size in chickens and ducks and plays a pivotal role in muscle growth and development (18–20). Notably, IGF2BP1 exhibits high expression levels during the early stages of muscle development but becomes nearly absent in normal adult muscles (21). Further investigation revealed a significant association between single nucleotide polymorphisms (SNPs) in the promoter region of the *IGF2BP3* gene and breast muscle weight. Furthermore, the mRNA expression level of *IGF2BP3* was remarkably higher in the pectoralis of high-body-weight chickens compared to those of low-body-weight chickens during both the embryonic stage and the post-hatch period (22). Therefore, it is reasonable to propose that the differential expression of *IGF2BPs* may contribute to breast muscle development.

Additionally, m6A methylation modifications are important for skeletal muscle development in ducks (23). IGF2BPs (IGF2BP1, IGF2BP2, and IGF2BP3) have been found to act as readers of a unique m6A family that regulates gene transcription through m6A methylation (24). *IGF2BP1* has been identified as a key gene regulating pig embryonic skeletal muscle development through m6A modification (25). In chicken myoblasts, the overexpression of IGF2BP1 alone inhibited proliferation, however, the co-expression of Mettl21c and IGF2BP1 abolished the inhibitory effects of IGF2BP1, thereby promoting cell proliferation and differentiation. Furthermore, it was observed that METTL21C could mediate the lysine methylation modification of IGF2BP1 (26). In this study, the m6A RNA methylase gene *METTL21C* was found to be up-regulated from D42 to D70 in female Muscovy ducks. The results indicate that *IGF2BPs* play a crucial role in the muscle development of Muscovy ducks through RNA methylation.

From day 28 to day 70, both male and female ducks consistently exhibited upregulation of the *KIT* and *MYLK4* genes in their breast muscles. The *KIT* gene is recognized as a target of the *MITF* gene, which may regulate muscle and fat production by influencing PGC-1 or MAPK1, ultimately affecting muscle performance and quality at slaughter (27). Notably, it is also hypothesized that the *KIT* gene influences muscle growth and development, which further impacts slaughter performance and muscle quality. This is consistent with our correlation analyses, which revealed that KIT was significantly positively correlated with myofiber diameter and cross-sectional area, while being significantly negatively correlated with myofiber density. MYLK4 plays a crucial role in the development of slow myofibers in pigs (28). Additionally, the knockout of MYLK4 in mice leads to a decrease in muscle stiffness and weight loss (29). In our analysis, we observed a consistent and significant increase in the expression of the *MYLK4* gene in the breast muscles of both male and female Muscovy ducks from day 28 to day 70. Our previous studies identified the peak period of breast muscle development in Muscovy ducks to be between days 42 and 70 (15). The increase in myofiber diameter and cross-sectional area during this critical period significantly contributes to the rate of muscle development (29). Furthermore, we found that the expression of *MYLK4* was significantly positively correlated with myofiber diameter and cross-sectional area, while being significantly negatively correlated with myofiber density. The heightened expression of the *MYLK4* gene aligns with the continuous growth of myofiber in terms of diameter. Overall, these findings suggest that the increased expression of both the *MYLK4* and *KIT* genes plays a pivotal role in the hypertrophy of myofiber in Muscovy ducks.

At D70, the myofiber characteristics and body weight showed significant differences between male and female Muscovy ducks, thus, we analyzed the DEGs at D70 to explore the causes of sexual dimorphism in Muscovy duck growth and development. We observed a remarkable reduction in *ANKRD1* expression in male pectoral muscles compared to female pectoral muscles at 70 days of age. ANKRD1 is a member of the muscle ankyrin repeat protein family, is highly expressed in less tender pork and has been linked to the woody meat formation in chicken breast muscle (30, 31). In Iberian Pigs, ANKRD1 has been identified as a molecular marker of meat tenderness (32). Previous research has shown that the expression of ANKRD1 in cultured myoblasts diminishes in the presence of testosterone (33). These findings imply that the expression of the *ANKRD1* gene in adult breast muscle may be influenced by sex hormones and is a key factor contributing to the differences in pectoral muscle weights between sexes. Therefore, the high expression of *ANKRD1* in female black Muscovy ducks may be related to testosterone inhibition, potentially reducing the tenderness of female pectoral muscles.

Concurrently, we screened three genes—*TPM2*, *VCP*, and *HNRNPK*—whose expressions were significantly higher in the breast muscle of female ducks than that of male ducks at 28, 42 and 70 days. TPM2 is the major myosin isoform in skeletal muscle and was enriched in the hypertrophic cardiomyopathy (HCM) signaling pathway in this study. It regulates the activity of ATPase and affects the sensitivity of myofilaments to calcium ions, thereby participating in muscle contraction and stabilizing the cytoskeletal structure. In addition, TPM2 is associated with a variety of muscle diseases. In particular, it has been identified as a fibroblast-specific biomarker in human tumor studies (34, 35). Studies have shown that estrogen can induce the expression of TPM2 gene. In myofibroblasts, downregulation of the *TPM2* gene leads to upregulation of Bax and downregulation of Bcl-2, thereby promoting apoptosis (36). Therefore, the upregulation of *TPM2* gene expression in all three stages in female Muscovy ducks compared with males may be attributed to estrogen, which further affects muscle development.

HNRNPK, a multifunctional protein involved in transcription, translation, and other cellular processes, regulates the eIF2α/Atf4 pathway in differentiated myoblasts. Mutations in the *HNRNPK* gene lead to a decrease in the cell proliferation, a drastic decrease in myosin heavy chain (MHC), and myotube defects, impacting myoblast proliferation and differentiation (37, 38). In male black Muscovy ducks, the expression of *HNRNPK* is significantly lower than that in females at three development stage, with further downregulated in male breast muscles from 42 to 70 days of age. In C2C12 cells, *HNRNPK* may promote proliferation or inhibit differentiation of by up-regulating c-Src and down-regulating the expression of the MRFs family of myogenic transcription factors (39), suggesting that the down-regulated of *HNRNPK* may be involved in the upregulated expression of myogenic transcription factors, which involved in the rapid breast growth and development in male black Muscovy ducks.

VCP is an ATPase associated with biological activities such as cell proliferation, differentiation and apoptosis, and inactivation of VCP results in impaired lysosomes and necrotic myopathy expression in mice (40). Studies have shown that VCP maintains myofibre integrity in normal human muscle tissue. Under conditions of muscle atrophy, knockdown of VCP increases the level of ubiquitination of myofibrillar proteins, thereby preventing muscle atrophy (41). In mice, VCP knockout resulted in changes in myofiber size accompanied by significant degeneration compared with wild type (40). Most of the impact of VCP on muscle properties and functions arises from its regulatory role in response to changes in expression, thus the lower expression of the *VCP* gene in the muscle of male ducks may significantly contribute to the observed differences in myofiber phenotype and muscle weight between the sexes.

Skeletal muscle growth and development is a complex process involving endocrine hormones, transcriptional regulation and signaling pathway factors. In our study, the enriched AMPK signaling pathway (*EEF2*, *PFKM*, *RAB11B*, *TBC1D1*, *CREB3*), ECM–receptor interaction (*CD36*, *DAG1*, *COL1A1*, *COL1A2*) and the calcium pathway (*ATP2A1*, *CAMK2*, *PLCD*) were significantly associated with muscle development. Previous studies have suggested that the activation level of AMP-activated protein kinase (AMPK) signaling pathway plays a significant role in the metabolic control of skeletal muscle by regulating various downstream targets. Consequently, it is crucial for the regulation of muscle growth, size, and hypertrophy (42, 43). The phosphorylated AMPK (P-AMPK) can transmit signals to CD36, which is essential for the transport of fatty acids into cells for their uptake and utilization, thus supporting muscle growth and regulating muscle size and hypertrophic potential (44). Research has shown that the lack of CD36 may impede cell cycle progression by lowering the levels of MyoD and Myf5, while concurrently facilitating cell cycle exit via the activation of myostatin. Moreover, the reduced expression of CD36 negatively affects skeletal muscle cell proliferation by obstructing the transition from the G0/G1 phase to the S phase, a process reliant on the cell cycle proteins D1 and CDK4 (45). Our study demonstrated that the levels of *CD36* expression were significantly positively correlated with myofiber diameter and cross-sectional area, while showing a significant negative correlation with density. Additionally, the *CD36* gene exhibited considerable upregulation in the breast muscle of Muscovy ducks between 42 and 70 days of age, regardless of sex. These results suggest that the AMPK signaling pathway, mediated by genes like *CD36*, is important in modulating the hypertrophy of pectoral myofibers in black Muscovy ducks.

Furthermore, phosphorylation of AMPK promotes calcium levels in the body (46). Studies in mice have shown that impaired expression of genes related to calcium signaling pathway leads to impaired proliferation and differentiation of muscle stem cells, which in turn leads to a reduction in the number of myofibers and the volume of muscle tissue, and affects the process of muscle development and regeneration (47). The sarcoplasmic/endoplasmic reticulum Ca^2+^ ATPases (ATP2As/SERCAs) is the main Ca^2+^ pump of myotube and young myofibers, which reduces the level of intracellular Ca^2+^ by accumulating Ca^2+^ into sarcoplasmic reticulum. In human related studies, Atp2a1/Serca1 was found to be highly expressed in fast contracting skeletal muscle; Atp2a2/Serca2 in slow contracting skeletal muscle, vascular myocytes and cardiac myocytes (48, 49). In addition, the study on mouse C2C12 myoblasts showed that the expression of Atp2a1/Serca1b was necessary for the proliferation of myoblasts and secondary myotube formation (50). An increased expression of *ATP2A1* was detected in both male and female ducks from d28 to d42, and the expression of *ATP2A1* was significantly positive correlated with myofiber diameter and significantly negative correlated with myofiber density in black Muscovy ducks. Thus, the upregulated expression of the *ATP2A1* gene may involved in the increase of muscle mass in black Muscovy ducks. ATP2A2/SERCE2a, exhibited reduced expression in the pectoral muscle of male ducks from 42 to 70 days. ATP2A2 is known to be associated with myofiber composition and is considered to be a vital gene for determining myofiber characteristics in chickens. It is highly expressed in the slow myofiber of cardiac and skeletal muscle (51). The activity of ATP2A2 intensifies with increasing adiposity, and ATP2A2 plays a role in regulating intramuscular fat deposition by enhancing new adipogenesis and inhibiting lipolysis through the involvement of Ca^2+^ (52). Furthermore, *ATP2A2* may play a role in regulating the muscle flavor and tenderness, its differential expression between males and females resulted in differences in myofibre development, ultimately causing differences in myofibre characteristics.

The ECM-receptor interaction play a crucial role in the development of duck muscle (12), and skeletal muscle extracellular matrix (ECM) can regulate satellite cells activity and renewal, influencing skeletal muscle tissue regeneration and repair (53). Collagen, an essential component of the ECM in skeletal muscle, plays a significant role in determining the physicochemical properties of muscle tissue and providing structural support (54, 55). In our investigation, collagen marker genes (*COL1A1*, *COL1A2*, *COL2A1*, *COL3A1*, *COL4A1*, *COL4A2*, *COL5A1*, and *COL6A1*) are DEGs enriched in the ECM-receptor interaction pathway, which are related to muscle development. From D42 to D70, both male and female ducks displayed a down-regulation of *COL1A1*, *COL1A2*, and *COL2A1*, and their expression was lower in females than in males. Previous studies have found that estrogen inhibits the proliferation of fibroblasts and the gene expression of type I-II collagen in female rats (56, 57), which may be a major contributor to the fact that breast muscle tenderness is not as good in female black Muscovy ducks as it is in males.

## 5 Conclusion

In summary, there was no significant difference in myofiber phenotypes between male and female ducks at 28 days of age, and the greatest difference was found at 70 days of age. Genes like *MYLK4*, *KIT*, *CD36*, *IGF2BP1*, *ATP2A1*, *TPM2*, *HNRNPK*, *VCP*, *ATP2A2*, and *ANKRD1* were identified as playing vital role in myofiber hypertrophy of black Muscovy ducks at different age and different sexes. Furthermore, key pathways such as AMPK signaling pathway, AGE-RAGE signaling pathway, ECM-receptor interaction cGMP-PKG signaling pathway, calcium signaling pathway, and hypertrophic cardiomyopathy were found to be significantly involved in myofiber development. The identified DEGs may serve as valuable molecular markers for selecting growth rate and meat production traits, enhancing production efficiency for ducks.

## Data Availability

The datasets presented in this study can be found in online repositories. The names of the repository/repositories and accession number(s) can be found in the article/Supplementary material.

## Footnotes

^1^ http://kobas.cbi.pku.edu.cn
