# Supplementary material for: Transcriptome analysis reveals the regulatory mechanism of myofiber development in male and female black Muscovy duck at different ages
Source: Front Vet Sci. 2024 Nov 20;11:1484102. doi: 10.3389/fvets.2024.1484102 (PMC11614779; doi:10.3389/fvets.2024.1484102)
Supplement: Supplementary file 1 [file Data_Sheet_1.docx]

Supplementary Material

# Supplementary Figures and Tables

## Supplementary Figures

**
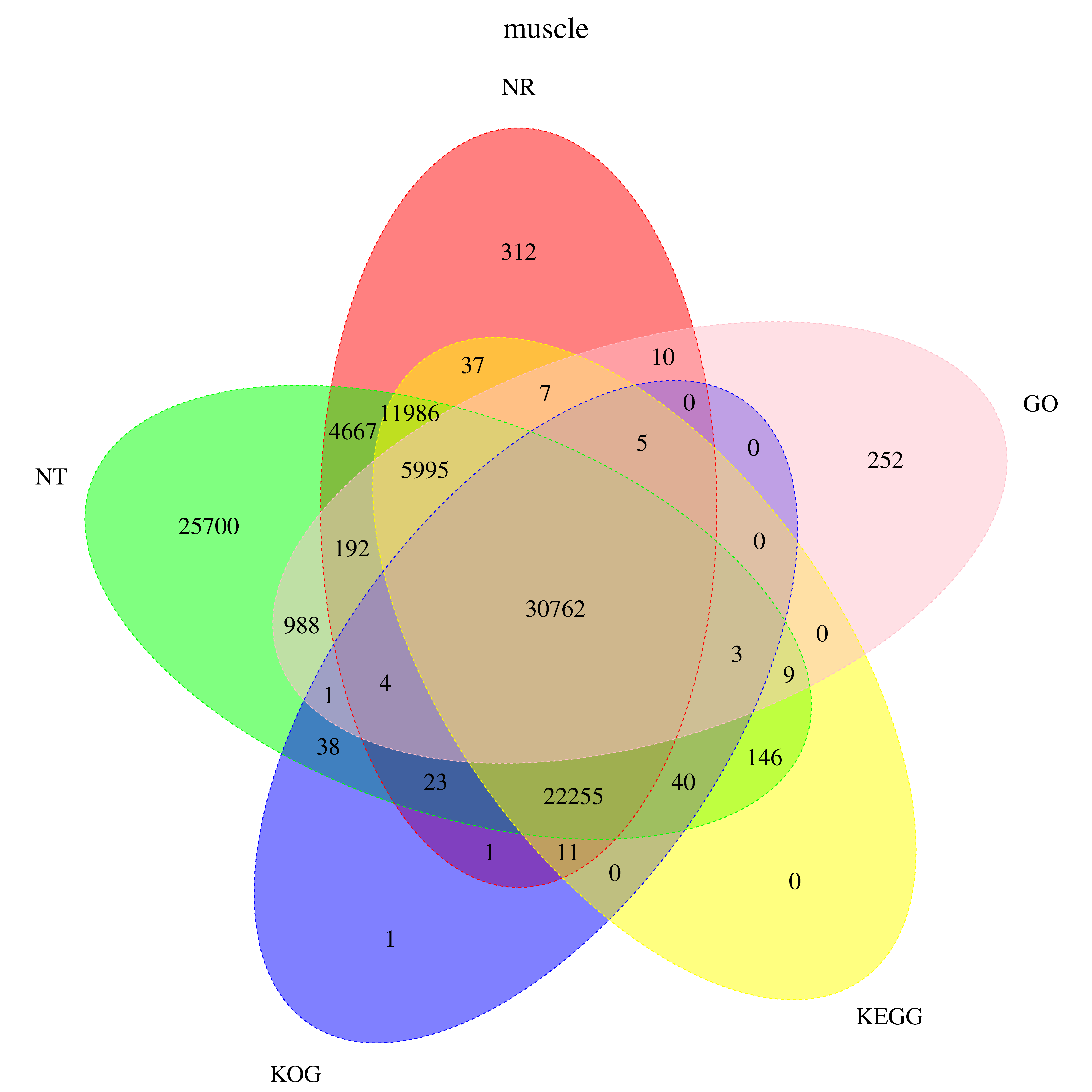
**

Supplementary Figure A1**.** Number of transcripts annotated to the five databases.

## Supplementary Tables

Table A1 Primer sequences of candidate differentially expressed genes for quantitative

| **Candidate differentially expressed** | | **Sequences** | **T/℃** |
| --- | --- | --- | --- |
| CD36 | Forward Primers(5'-3') | CATCTGAGTGGCCTCTGTCC | 60 |
|  | Reverse Primers(5'-3') | TCCCAACCCTTATTCCATCA | 60 |
| IGF2BP1 | Forward Primers(5'-3') | CCGCATTCCTTCCCCTCTAC | 60 |
|  | Reverse Primers(5'-3') | CCTTTGCCAATGACCCTCCC | 60 |
| IGF2BP2 | Forward Primers(5'-3') | GCAGTGGGAGGTGCTGGATG | 60 |
|  | Reverse Primers(5'-3') | GTGGCCGCTGAGCTTCTCAA | 60 |
| IGF2BP3 | Forward Primers(5'-3') | TGCTCACCACCCGTCTCTCT | 60 |
|  | Reverse Primers(5'-3') | AGAAGGCAGCAGCAACAGCA | 60 |
| SRF | Forward Primers(5'-3') | CTGTTCCGTCACCGCTGTTG | 60 |
|  | Reverse Primers(5'-3') | AGTAGAGCCCAGGGCGTCAG | 60 |
| FABP3 | Forward Primers(5'-3') | GCACGTCAAGTCCGTGGTCA | 60 |
|  | Reverse Primers(5'-3') | TGCCCATGGTGAGGGTCAGA | 60 |
| HSP90B1 | Forward Primers(5'-3') | TCAGCATCAGCATCCACCTC | 60 |
|  | Reverse Primers(5'-3') | GCTGAGGCGAGTCAAGGAAA | 60 |
| HSP90AA1 | Forward Primers(5'-3') | GGCAGAAGGCAGAGGCTGAC | 60 |
|  | Reverse Primers(5'-3') | TGCGGTTGGCATGTGTCTGA | 60 |
| MSH2 | Forward Primers(5'-3') | CAGAGTTGGCGGCTTTCCCA | 60 |
|  | Reverse Primers(5'-3') | TGGATGGCGGTTCTCCTTCA | 60 |
| MSTN | Forward Primers(5'-3') | GGCAATGCCTAGCGCACGTA | 60 |
|  | Reverse Primers(5'-3') | AGATCGTTGCGGCTGCTCAT | 60 |
| TGFB3 | Forward Primers(5'-3') | ACAGGACCTGGGCTGGAAGT | 60 |
|  | Reverse Primers(5'-3') | GCCTCAGGGTTCAGCGTGTT | 60 |
| THRAP3 | Forward Primers(5'-3') | AGCCCTCTCCAGTCGGTTGT | 60 |
|  | Reverse Primers(5'-3') | GGTGAGAGCCCGCCTGAAAG | 60 |
| FGF13 | Forward Primers(5'-3') | AGCCACCACCAACTGTCTGC | 60 |
|  | Reverse Primers(5'-3') | TACGCTGCACAGAGCATGGG | 60 |
| ANKRD1 | Forward Primers(5'-3') | CTCTGCACGTCGCTGTGAGG | 60 |
|  | Reverse Primers(5'-3') | CGGCGTCATGCATTGGTGTG | 60 |
| ATP2A1 | Forward Primers(5'-3') | GCGAGCAGTAGACCGACATG | 60 |
|  | Reverse Primers(5'-3') | CCTGCCTGGTGGAGAAGATG | 60 |
| ATP2A2 | Forward Primers(5'-3') | TTAGGGTTGCGGGTTGGCTT | 60 |
|  | Reverse Primers(5'-3') | TGACTGCTGCCCTGGGTTTC | 60 |
| MYLK4 | Forward Primers(5'-3') | GCAACTGGGCAGGGTGCTAA | 60 |
|  | Reverse Primers(5'-3') | CCAACTGTCCCATCCCTGCT | 60 |
| kit | Forward Primers(5'-3') | GCCCAGGAACTGAGCAGAGG | 60 |
|  | Reverse Primers(5'-3') | GTGCCGGTGCTCCTGAACAT | 60 |
| HPRT1 | Forward Primers(5'-3') | AATGGATAGTTGCACCTTTGGTTGTT | 60 |
|  | Reverse Primers(5'-3') | ACAGCGATAAGGCCACTCCA | 60 |
